# Modelo cuantitativo para mejorar el financiamiento de la atención primaria en Chile

**DOI:** 10.26633/RPSP.2017.173

**Published:** 2017-12-26

**Authors:** Rubén Estanislao Castro, Alain Palacios, Andrea Arenas, Bernardo Martorell

**Affiliations:** 1 Universidad Diego Portales Universidad Diego Portales Santiago de Chile Chile Universidad Diego Portales, Santiago de Chile, Chile.; 2 Ministerio de Salud de Chile Ministerio de Salud de Chile Chile Ministerio de Salud de Chile, Chile.

**Keywords:** Equidad en salud, sistema de pago prospectivo, administración financiera, Health equity, prospective payment system, financial management, Equidade em saúde, sistema de pagamento prospectivo, administração financeira

## Abstract

**Objective.:**

Propose and apply a methodology to estimate adjusted expected expenditure in each locality in the Chilean primary health care (PHC) system in 2016.

**Methods.:**

First of all, expected per capita expenditure at the national level was calculated on the basis of a detailed health plan, and then a zero-sum adjustment was made to the expenditure in each locality, using the local age/sex profile and the local average socioeconomic level, years of life lost, and rurality, given their statistically significant impact on epidemiology and spending structures.

**Results.:**

The model establishes a conceptual and empirical link between expected expenditure and adjustment variables; it is flexible in terms of successive improvements; and its zero-sum property facilitates discussion of the global budget. When real data for the year 2016 in Chile were used, it was found that the absolute distance between the amounts in the model and the amounts actually used that year was 7.6%, on average.

**Conclusions.:**

There are simple empirical options for calculating expected expenditure across localities, for which it is very helpful to have a good estimate of expected expenditure at the national level.

## RESUMEN

**Objetivo.:**

Proponer y aplicar una metodología para estimar el gasto esperado ajustado de cada localidad en el sistema chileno de atención primaria de la salud (APS) en el año 2016.

**Métodos.:**

En primer lugar, se formalizó el cálculo del gasto esperado per cápita a nivel nacional, en base a un detallado plan de salud, y luego se introdujo en este gasto un ajuste de suma cero para cada localidad a través del perfil local de sexo y edad y el promedio local del nivel socioeconómico, años de vida perdidos y ruralidad, por su incidencia estadísticamente significativa en las epidemiologías y en las estructuras de gasto.

**Resultados.:**

El modelo logra un vínculo conceptual y empírico entre los gastos esperados y las variables de ajuste, es flexible a mejoras sucesivas, y la propiedad de suma cero facilita la discusión del presupuesto global. En la aplicación a datos reales de Chile para el año 2016 se encuentra que la distancia absoluta entre los montos del modelo y los montos usados en dicho año es de 7,6% en promedio.

**Conclusiones.:**

Existen alternativas sencillas para sustentar empíricamente el cálculo de los gastos esperados a través de las localidades, para lo cual es de gran ayuda contar con una buena estimación del gasto esperado a nivel nacional.

**Objetivo.:**

Propor e implementar uma metodologia para o cálculo do gasto com saúde projetado ajustado para cada municipalidade no sistema chileno de atenção primária à saúde para o ano de 2016.

**Métodos.:**

Primeiro padronizou-se o cálculo do gasto per capita projetado em nível nacional, baseado em um detalhado plano de saúde. Em seguida foi feito um ajuste de soma zero no gasto com saúde para cada municipalidade segundo o perfil local de sexo e idade da população e o valor médio para nível socioeconômico, anos de vida perdidos e ruralidade de acordo com a associação de significância estatística em estudos epidemiológicos e estruturas do gasto.

**Resultados.:**

O modelo consegue estabelecer um vínculo conceitual e empírico entre os gastos projetados e as variáveis de ajuste, ele se adapta a melhorias sucessivas e a propriedade de soma zero facilita a discussão do orçamento global. Ao serem aplicados dados reais do Chile para 2016, observou-se uma distância absoluta entre os valores do modelo e os valores do ano considerado de, em média, 7,6%.

**Conclusões.:**

Existem alternativas simples para fundamentar empiricamente o cálculo do gasto com saúde projetado para as municipalidades, sendo bastante útil poder dispor de uma boa estimativa do gasto com saúde projetado em nível nacional.

La atención primaria de salud (APS) es un pilar fundamental de la salud pública de un país; aparece ligada a menores gastos, menos hospitalizaciones y menos uso de servicios de emergencia (1, 2). Además, atenúa el impacto de la condición socioeconómica en la salud (3). En el caso de Chile, de hecho, el financiamiento de este pilar ha mostrado un crecimiento mayor al de la atención en los niveles secundario y terciario (4).

En el nivel internacional, hay una clara tendencia hacia el financiamiento per cápita de la APS (5), en especial allí donde el Estado provee el servicio de salud primaria (6). El uso de la capitación normalmente va acompañado de una metodología de tipo ajuste de riesgo (*risk adjustment*) para ajustar el financiamiento per cápita global a las condiciones de cada localidad (7-9). Una descripción general sobre la teoría y la práctica de las metodologías de ajuste en el financiamiento de la salud en el mundo se expone en Rice y Smith (2001) (7), probablemente el principal artículo científico en la materia. En este artículo se explica que, dado un presupuesto total fijado en forma exógena, se seleccionan variables de ajuste y se establece el peso de cada una de ellas en el gasto esperado per cápita promedio.

El espíritu de la metodología del ajuste, entonces, es de suma cero, es decir su aplicación no altera el gasto esperado a nivel nacional^3^, como se destaca en la literatura que compara distintos sistemas a través del mundo (6, 7, 10, 11). La idea es tomar un gasto esperado per cápita a nivel global y luego ajustarlo a las condiciones relativas de cada localidad, pues determinadas localidades resultan tener, en algunas dimensiones, una posición ventajosa o desventajosa en los gastos esperados en la APS. Subyace a ello el ánimo de lograr una distribución de los recursos más equitativa en el sentido de personas con similares necesidades y similar acceso al sistema; este principio se ha denominado “horizontal” y es el más común en los sistemas de salud (7, 12, 13).

Pero en la práctica, para definir el impacto cuantitativo de cada variable de ajuste, se utiliza el impacto de estas en los gastos observados o en las tasas de uso observadas. Sin embargo, ello solo replica la situación actual, con lo que puede premiar la ineficiencia, introducir incentivos perversos al dar a los administradores herramientas para aumentar su financiamiento en forma oportunista y, además, debilita el vínculo conceptual entre las variables de ajuste y el gasto esperado.

Entre las excepciones, el sistema británico utiliza un modelo para estimar el ajuste de los gastos esperados per cápita a nivel nacional a las condiciones de cada localidad (9, 14, 15), aunque la mayoría de los países no han incorporado este enfoque en sus sistemas de salud. De manera progresiva, la capitación en salud primaria comenzó a ser utilizada en varios países de América Latina (Brasil, Bolivia, Chile, Colombia, México, Suriname y Uruguay) (6, 16), pero las metodologías de ajuste del financiamiento per cápita se concentran en gastos observados o en reglas de eficiencia. En Chile se utiliza una estimación cualitativa del ajuste, sin la propiedad de suma cero y sin un respaldo escrito disponible que justifique el impacto cuantitativo de cada variable de ajuste; este sistema ha permanecido sin cambios desde 1994 a la fecha. Varios autores han analizado la posibilidad de mejorar algunas variables de ajuste (17-22), mas no han formalizado una metodología de estimación del ajuste. Dado el interés en el aporte de la APS a la equidad que se observa en la región (12), se hace recomendable estudiar alternativas metodológicas que faciliten una distribución equitativa del financiamiento total.

La literatura menciona una serie de elementos deseables en este tipo de ajustes en el financiamiento de la APS, resumidos en Smith (9): deben ser factibles, con bajo gasto de administración, consistentes (que tienden al “verdadero valor”), confiables (que arrojan resultados muy similares si se repite el ejercicio), certificables, construidos para todas las localidades o zonas administrativas, no vulnerables a ser manipulados, válidos (que resulten, de manera legítima, un buen predictor de los gastos esperados), que incentiven a la eficiencia y libres de incentivos perversos.

Con base en estas recomendaciones, el objetivo de este estudio es proponer y aplicar una metodología para estimar el gasto esperado ajustado de cada localidad en el sistema chileno de APS en el año 2016. El sistema chileno cuenta con un detallado plan de APS, cuya matriz de costeo se formaliza en este estudio y luego se propone, sobre ella, una selección de variables de ajuste y una metodología de estimación de su impacto cuantitativo. El modelo se aplica a todo el sistema (275 localidades), si bien no se incluyen localidades de menos de 3 500 habitantes (que se financian con un aporte fijo y en total representan 0,001% del financiamiento total del APS), y tampoco se incluye la APS administrada en el nivel central, que representa aproximadamente 8% del presupuesto total de la APS en Chile. Tampoco se incluyen los aportes por “asignación de zona” y “desempeño difícil”, cuya naturaleza normativa se aleja del concepto de gasto esperado y, de hecho, proviene de regulaciones que afectan a toda la administración pública chilena.

## MATERIALES Y MÉTODOS

La propuesta se basa en la existencia de una matriz de costeo del plan de APS, la cual se formaliza a continuación.

### Formalización de la metodología actual de costeo del plan de APS

En Chile, el plan de APS consiste principalmente en un conjunto de actividades llamadas Plan de Salud Familiar y financiadas mediante un aporte prospectivo per cápita. Este monto corresponde, desde el punto de vista conceptual, al gasto esperado per cápita. El plan de salud familiar contiene unas 150 actividades, que cada localidad debe administrar en su población de usuarios inscritos. Por ejemplo, la actividad “control de malnutrición en población infantil” define cuatro atenciones anuales para los niños menores de seis años en riesgo de malnutrición. En general, el plan de salud familiar es un conjunto de prestaciones de promoción, prevención, tratamiento y rehabilitación.

La ecuación 1 formaliza el cálculo del gasto mensual del plan APS:

**Figure:**
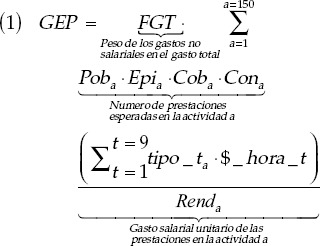


donde

GEP: gasto esperado per cápita mensual del plan de APS a nivel nacional.

FGT: factor de gastos totales. Este corresponde a un amplificador que da cuenta de todos los otros gastos distintos del gasto salarial del recurso humano de salud. No incluye inversión e infraestructura.

Pob_a_: proporción de la población total en el sistema APS, para la cual se define la actividad *a* (por ejemplo, 5,6% de la población total correspondería a niños menores de seis años).

Epi_a_: tasa epidemiológica en la actividad *a* (por ejemplo, 10% de la población de niños menores de seis años presentaría malnutrición).

Cob_a_: cobertura en la actividad *a*, porcentaje de la población que se pretende cubrir.

Con_a_: concentración mensual en la actividad *a*, corresponde al número de prestaciones anuales por caso en la actividad *a*, divididas por doce.

t: tipo de recurso humano de salud: médico, enfermero, odontólogo, sicólogo, nutricionista, kinesiólogo, paramédico, asistente social o matrona.

tipo_t_,a_: participación de un profesional tipo *t* en el conjunto de recursos humanos de salud que se utiliza para otorgar una prestación en la actividad *a*.

Rend_a_: rendimiento en la actividad *a*, número de atenciones por hora que logra el conjunto de personal de salud en la actividad *a*.

$_hora_t: corresponde al salario por hora del personal de salud tipo *t*.

El FGT proviene de la naturaleza de la APS, donde buena parte de los gastos corresponden al recurso humano. Ahora bien, en su sencillez, resulta difícil justificar con precisión un valor por sobre otro, si bien versiones futuras de la ecuación 1 debieran profundizar el modelamiento de este factor. En el caso de Chile, este se ha fijado tradicionalmente muy en torno a “2”. Cabe notar que la ecuación 1 solo define el financiamiento total de cada localidad, aunque estas se administran en forma autónoma y, por ello, el FGT “efectivo” es distinto en cada localidad.

### Metodología propuesta para el ajuste del GEP

El objeto de la propuesta es contar con una estimación del GEP ajustado a las condiciones de cada localidad. Cada uno de los tres elementos en la ecuación 1 (factor de gastos totales, cantidad de prestaciones y gasto salarial unitario) presenta una oportunidad para ajustar el GEP a las condiciones de cada localidad. En virtud de las variables disponibles y las recomendaciones en la literatura (7), este estudio propone cinco variables de ajuste de los GEP, que pueden clasificarse en tres grupos: demográfico, epidemiológico y geográfico.

#### Ajuste demográfico.

El perfil de sexo y edad en los gastos esperados per cápita está presente en la gran mayoría o todos los sistemas de salud, y se corresponde con el perfil clásico de mortalidad y fecundidad. El ajuste del GEP a las condiciones demográficas de una localidad en particular se configura aquí reemplazando el perfil de sexo y edad nacional en la ecuación 1 por el perfil de la localidad. De esta forma, el impacto del perfil de sexo y edad en el GEP aparece de manera directa desde de la matriz de costeo del plan de APS y ello arroja un GEP ajustado por sexo y edad.

#### Ajuste epidemiológico.

Cada actividad en el plan de APS está ligada a una prevalencia o incidencia de alguna condición de salud. Para estimar el impacto cuantitativo de las variables de ajuste en la epidemiología, se propone, por simpleza, estimar el impacto porcentual de las variables de ajuste en la salud general y luego aplicar ese mismo impacto a todas las prevalencias en el plan de APS. Para ello, se propone analizar desde un punto de vista econométrico el impacto que las variables de ajuste tienen en una pregunta de salud general en la encuesta de Caracterización Socioeconómica Nacional (CASEN) 2013. Versiones futuras de esta metodología pueden estimar el impacto de las variables de ajuste directamente en la prevalencia relevante, actividad por actividad, mediante el uso de la Encuesta Nacional de Salud u otras fuentes de información.

Dentro de las variables disponibles, este estudio ha seleccionado los años de vida potencialmente perdidos (AVPP) por estar disponible a nivel local, en forma oficial y actualizada, y porque puede representar en forma agregada la situación profunda del estado de salud de las personas, independiente de la configuración local de la APS. Esto dificulta que los administradores locales influyan en ella, salvo en el muy largo plazo. Los AVPP suelen ser utilizados como indicador epidemiológico en el sistema chileno de salud. Además, se ha seleccionado la variable “proporción de la población inscrita en los dos tramos más bajos de salario del sistema público de salud” como indicador socioeconómico (ISE) por estar disponible a nivel local, en forma oficial y actualizada, y por el reconocido vínculo entre la epidemiología y el contexto socioeconómico. No se utilizaron variables de uso de servicios o registros locales de tasas de prevalencia por estar ligadas en forma directa a la gestión de los administradores locales. Las otras variables socioeconómicas que se analizaron en este estudio fueron la “proporción de la población inscrita en el tramo más bajo de salario del sistema público de salud”, el “índice de privación promedio municipal”, el “índice de inequidad territorial de género” y el “índice de vulnerabilidad”, aunque no resultaron estadísticamente significativas. La ecuación propuesta para estimar el ajuste a las epidemiologías del plan de APS es:

$$(2)\;\hat{EPI_{x}}=1+b_{1}\cdot \hat{ISE_{x}}+b_{2}\cdot \hat{AVPP_{x}}+e_{x}$$

donde EPI_x_ corresponde a la tasa epidemiológica de la localidad x, b_1_ y b_2_ son las elasticidades de la epidemiología con respecto al indicador socioeconómico (ISE) y los años de vida perdidos (AVPP), y el símbolo de un techo corresponde a las variables expresadas en “valor con respecto a la media nacional”. De esta forma, los coeficientes b_1_ y b_2_ representan la elasticidad de las tasas epidemiológicas con respecto a ISE y AVPP, respectivamente^4^. Por último, la ecuación 2 se estima utilizando la variable dicotómica de CASEN 2013 “autorreporte de enfermedad o accidente en los últimos tres meses” como variable dependiente, de donde se obtiene la estimación de las elasticidades.

#### Ajuste de la función de gastos.

Las condiciones del entorno constituyen uno de los espacios de ajuste más comunes en los sistemas de financiamiento de APS (7), debido a que los gastos medios de producción son mayores en localidades donde la población se ubica en forma dispersa y/o las condiciones geográficas son difíciles. El gasto en ambulancias, la cadena de frío en exámenes, los viajes del personal de salud y la realización de rondas médicas serían ejemplos de funciones productivas que se encarecen o abaratan cuando la condición sociogeográfica de una localidad es más o menos adversa que el promedio nacional.

Las posibles variables de ajuste disponibles son la ruralidad^5^ y la densidad poblacional^6^, sin tener en cuenta indicadores de aislamiento porque su metodología de cálculo está en proceso de desarrollo y, además, no se tiene una periodicidad programada para actualizarlos^7^.

La ecuación 1 ofrece cuatro dimensiones para ajustar los gastos medios de producción: el rendimiento del conjunto del personal de salud en cada actividad, la composición del conjunto del personal de salud en cada actividad, el precio por hora de cada tipo de personal de salud y el factor de gastos totales. De las cuatro alternativas, el rendimiento y la composición del conjunto del personal de salud no parecen relacionarse, desde un punto de vista conceptual, con las dinámicas de gasto que se desea ajustar, y el precio por hora no muestra una asociación estadísticamente significativa con la ruralidad y densidad^8^. El FGT, por otra parte, ofrece una alternativa conceptual coherente con la intuición y además muestra una asociación estadísticamente significativa con las variables de ajuste. Ahora bien, en la actualidad no se cuenta con los datos de FGT por localidad en el sistema chileno, por lo que este se ha estimado en este estudio mediante datos contables y datos de salarios de personal de salud en el sistema APS.

Para estimar la elasticidad del FGT local a la ruralidad local, se ha utilizado la siguiente regresión lineal:

$$(3)\;{\hat{FGT}}_{x}=1+a_{1}*\hat{RUR_{x}}+e_{x}$$

donde todas las variables se miden en desviación sobre la media nacional y a_1_ corresponde a una elasticidad. Debido, tal vez, a la colinealidad entre ruralidad y densidad, esta última no muestra significancia estadística como variable de ajuste, por lo que se ha mantenido solo la ruralidad.

En suma, la metodología propuesta consiste en la formalización de la matriz de costeo de la APS definida en la ecuación 1, la estimación de las elasticidades necesarias para el ajuste epidemiológico y de la función de gastos y el cálculo del ajuste del GEP ajustado de cada localidad, el cual va multiplicado por una constante para asegurar que el ajuste sea de suma cero. Además, por último, existe la opción de expresar el valor ajustado como el resultado de un polinomio, con el objetivo de facilitar el uso como herramienta de política pública; los coeficientes de cada variable de ajuste podrían estimarse mediante un modelo estadístico que minimice la distancia entre el valor del modelo y el valor del polinomio.

## RESULTADOS

La metodología propuesta se aplicó a datos reales del año 2016. De acuerdo a los datos, el GEP en dicho año fue de pesos chilenos (CLP) 5 420 mensuales, es decir en torno a nueve dólares^9^. Para replicar dicho valor, se mantuvieron constantes todos los parámetros del costeo del plan de salud de APS, excepto los precios por hora del personal de salud, los cuales fueron amplificados de manera uniforme. Las elasticidades estimadas se detallan en el cuadro 1.

**CUADRO 1.** Elasticidades de la epidemiología y el factor de gastos totales con respecto a sus determinantes

	Número de localidades: 278		
	Promedio	Desviación estándar	Elasticidad estimada (regresiones 2 y 3)^a^
ISE^b^	56%	8%	0,54
AVPP	80	18%	0,16
Ruralidad^c^	34%	28%	0,22

AVPP, años potenciales de vida perdidos por cada mil habitantes; ISE, indicador socioeconómico.

a Todos los valores anteriores son estadísticamente significativos.

b Porcentaje de la población inscrita que está en los tramos de salario más bajos (A y B).

c Nótese que la ruralidad nacional de Chile es 11%, mucho más baja que 34%, el promedio simple de las localidades.

**FIGURA 1.**
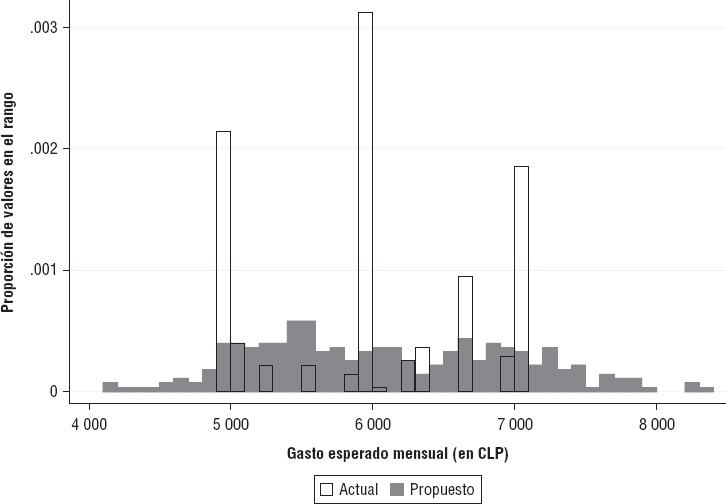
Histograma del gasto esperado promedio ajustado: valor actual de 2016 y valor propuesto

Los GEP ajustados que arroja el modelo tienen una desviación estándar de CLP 896, algo mayor a la observada en los valores actuales del año 2016, CLP 743 y, en línea con ello, muestran un mayor valor máximo y menor valor mínimo. Además, la distribución es mucho más normal en el modelo propuesto, reflejando la distribución normal de las localidades con respecto al promedio nacional en cada variable de ajuste (figura 1).

***Fuente:*** Elaboración del autor.

CLP, pesos chilenos.

La distribución de las diferencias entre el valor propuesto y el real se ubica entre -10% y +10% para la mayoría de las localidades; la distancia absoluta entre los montos del modelo y los montos usados en dicho año es de 7,6% en promedio. Por ello, una transición entre el modelo actual y el modelo propuesto en este estudio puede ser suavizada con una cantidad muy moderada de recursos extra.

La equidad, por otra parte, medida por la correlación entre el valor del modelo y los indicadores socioeconómicos locales disponibles (índice de privación promedio municipal, índice de inequidad territorial de género, índice de vulnerabilidad e índice de desarrollo humano), es muy parecida a la observada en los valores reales del 2016.

Por último, la expresión del valor ajustado como el resultado de un polinomio (el ajuste demográfico se resumió en una única variable: “porcentaje de adultos de 65 y más años” porque el porcentaje de mujeres no resultó significativo, tal vez por su poca varianza a través de las localidades) se muestra en el cuadro 2.

**CUADRO 2.** Gasto esperado de cada comuna expresado como una función lineal de las variables de ajuste

	Coeficientes del polinomio	Coeficiente beta para comparación
Indicador socioeconómico	$36	0,332
Años de vida potencialmente perdidos	$7	0,180
Ruralidad	$19	0,592
Porcentaje de personas de 65 años y más	$50	0,154
Constante	$2 292	ND
Observaciones	278	278
R^2^ (coeficiente de determinación)	0,996	0,996

***Fuente:*** Elaboración del autor.

Todos los valores anteriores son estadísticamente significativos al 1%. Los aportes por “asignación de zona” y “desempeño difícil” no se incluyen en este estudio.

ND, no disponible

Los resultados que se muestran en el cuadro 2 son todos significativos, con un valor de *P* menor a 1%; además, el R^2^ de la regresión es muy alto (0,996), lo cual indica que los valores del modelo son “linealizables” en un polinomio. El polinomio entonces arroja un valor uniforme de CLP 2 292 mensuales por localidad, más CLP 36 por cada punto porcentual de población local inscrita en los dos tramos bajos de salario del sistema público de salud, más CLP 7 por cada año de vida potencialmente perdido por cada mil habitantes, más CLP 19 por cada punto porcentual de población rural, más CLP 50 por cada punto porcentual de población con 65 o más años.

Si bien los resultados parecen sugerir que el ajuste por edad es el más importante, la influencia de cada ajuste también depende de cuán dispersas son las variables de ajuste. Para comparar el impacto de cada variable de ajuste en el polinomio, se realizó un análisis de coeficientes beta, que consiste en estandarizar las variables restándoles su media y dividiéndolas por su desviación estándar, de forma que la influencia de cada variable pueda compararse razonablemente por la vía de comparar los coeficientes. En el cuadro 2 se muestra los coeficientes beta, donde se aprecia que la variable de ajuste más influyente es la ruralidad seguida por el indicador socioeconómico.

## DISCUSIÓN

La capitación del financiamiento de la atención primaria de salud es recomendada para controlar el gasto total y apoyar la equidad (7). En la capitación suele ajustarse un valor promedio a las condiciones de cada localidad. En las comparaciones de modelos en la literatura, (6, 7, 10, 11) se aprecia con claridad que existe una amplia gama de aproximaciones metodológicas, pero la mayoría de ellas se basa en los gastos observados, lo que introduce una suerte de circularidad en el ajuste. En general, las condiciones de cada país influyen en la configuración de su enfoque metodológico. En América Latina, la evidencia disponible indica que se utilizan ajustes basados en gastos observados.

En este trabajo se obtiene y aplica un marco conceptual y empírico para la estimación de un ajuste de los gastos esperados a la situación de cada localidad administradora en el sistema de atención primaria de salud en Chile. A diferencia del modelo actual, el modelo propuesto se desprende de un marco conceptual y empírico que acerca la distribución del financiamiento a la distribución de los gastos esperados por localidad, en línea con el comúnmente llamado principio de equidad horizontal. Además, en consonancia con las recomendaciones y experiencia internacional (por ejemplo, el sistema inglés y el sistema colombiano), el ajuste propuesto no altera el gasto nacional, es de “suma cero”, por lo que no necesita ser discutido a nivel de presupuesto central. Por otra parte, los valores ajustados generados con el modelo propuesto se hallan cerca de la estructura de financiamiento actual (como era de esperar si esta guarda alguna relación con los gastos esperados), lo que facilita la transición desde el sistema actual al sistema propuesto. La variable más influyente en el ajuste resultó ser la ruralidad, seguida por el indicador socioeconómico, luego el indicador de mortalidad y el indicador de adultos mayores.

Una de las lecciones generales que deja este estudio es la dificultad de contar con variables que sean medidas oficiales y que estén disponibles de manera regular para todas las localidades administradoras.

Para el modelo propuesto, así como para la discusión presupuestaria y administrativa, es central la existencia de un plan claro y formal que defina al sistema de APS. Aun así, debe tenerse en cuenta que cada unidad de administración está sujeta a riesgos de todo tipo, sobre todo las más pequeñas (cambios en la salud de la población, variaciones en la disponibilidad de recursos humanos y otros) y que, además todo modelo de financiamiento tiene sus imperfecciones. De allí que la cercanía entre el plan de APS (y, por lo tanto, el ajuste propuesto en este estudio) y la realidad puede ser variable y ello requiere poner atención en la conexión entre ellas.

Dentro de las limitaciones del estudio, además de las desventajas propias del análisis estadístico y de la calidad de los datos, cabe mencionar el problema de validez que, en mayor o menor grado, está presente en todas las metodologías de ajuste mencionado en la literatura. De todas formas, en el modelo propuesto el ajuste se apoya en el diseño teórico del plan de APS, lo que suaviza este problema. El ajuste epidemiológico, en particular, se basa en una regresión con datos individuales, lo que reduce el problema de validez; por último, el ajuste de la función de gastos no se enfoca en el nivel del gasto, sino en su estructura, la cual es relativamente exógena a la estructura del financiamiento capitado. Otra limitación es la simpleza del factor de gastos totales con que se costea el plan de APS, la cual limita la profundidad con que se puede ajustar dicho factor.

Por último, es importante señalar que una linealización puede facilitar el uso de la metodología propuesta, pero deben evitarse interpretaciones erróneas de los parámetros.

## Agradecimientos.

Los autores agradecen el valioso aporte de David Debrott para la metodología del cálculo del gasto esperado per cápita a nivel nacional, y también a Carla Castillo y a dos revisores anónimos por sus valiosos comentarios.

## Declaración.

Las opiniones expresadas en este manuscrito son responsabilidad del autor y no reflejan necesariamente los criterios ni la política de la *RPSP/PAJPH* y/o de la OPS.

## Footnotes

3 Esta propiedad se deriva tanto de la sola existencia de presupuestos globales que anteceden al papel de los ajustes, como de la idea de ajustar con respecto a un punto de referencia, pues un punto de referencia distinto del promedio nacional puede generar arbitrariedades metodológicas e inestabilidad en el recalculo periódico de los parámetros. El sistema en uso en Inglaterra, comúnmente visto como un modelo para los demás países, oficialmente utiliza ajustes de suma cero, así como Colombia y otros países. El Banco Mundial también ha recomendado esta propiedad.

4 Un ISE 10% menor a la media nacional, por ejemplo, se asociaría con una EPI 10%* b1 menor a la media nacional.

5 Se entiende como entidad rural a aquellos asentamientos humanos concentrados o dispersos en el que habitan menos de 2 000 personas, con menos de 50% de su población económicamente activa dedicada a actividades secundarias y/o terciarias (INE, 2004).

6 Población local sobre superficie de la localidad expresada en kilómetros cuadrados.

7 Además, sería posible explorar el gasto de exámenes y farmacia. En la actualidad, estos se incluyen en el FGT pero podrían ser relacionados directamente a cada actividad en el plan de salud de APS. Por razones de alcance en este estudio no se ha profundizado en esta alternativa.

9 Este valor no incluye las asignaciones de zona y por desempeño difícil, como se mencionó antes y la cotización del dólar estadounidense corresponde al día 4 de noviembre de 2017.
